# Quantitative analysis of prompt gamma ray imaging during proton boron fusion therapy according to boron concentration

**DOI:** 10.18632/oncotarget.23201

**Published:** 2017-12-14

**Authors:** Han-Back Shin, Moo-Sub Kim, Sunmi Kim, Kyu Bom Kim, Joo-Young Jung, Do-Kun Yoon, Tae Suk Suh

**Affiliations:** ^1^ Department of Biomedical Engineering and Research Institute of Biomedical Engineering, College of Medicine, The Catholic University of Korea, Seoul, Republic of Korea; ^2^ Department of Electronic Engineering, Sogang University, Seoul, Republic of Korea; ^3^ Department of Radiation Oncology, University of Florida, Gainesville, FL, USA

**Keywords:** proton boron fusion therapy, prompt gamma ray image, boron concentration, monte carlo simulation, tumor monitoring technique

## Abstract

The purpose of this study is to evaluate the prompt gamma ray imaging technique according to the clinical boron concentration range during proton boron fusion therapy (PBFT). To acquire a prompt gamma ray image from 32 projections, we simulated four head single photon emission computed tomography and a proton beam nozzle using a Monte Carlo simulation. We used modified ordered subset expectation maximization reconstruction algorithm with a graphic processing unit for fast image acquisition. Boron concentration was set as 20 to 100 μg at intervals of 20 μg. For quantitative analysis of the prompt gamma ray image, we acquired an image profile drawn through two boron uptake regions (BURs) and calculated the contrast value, signal-to-noise ratio (SNR), and difference between the physical target volume and volume of the prompt gamma ray image. The relative counts of prompt gamma rays were noticeably increased with increasing boron concentration. Although the intensities on the image profiles showed a similar tendency according to the boron concentration, the SNR and contrast value were improved with increasing boron concentration. This study suggests that a tumor monitoring technique using prompt gamma ray detection can be clinically applicable even if the boron concentration is relatively low.

## INTRODUCTION

The purpose of radiation therapy is delivery of a high dose to the tumor region, while minimizing the irradiation of healthy tissue. The kind of particle beam is proton, neutron, heavy ion beam, etc. Proton beam have the characteristic of rapid energy loss in specific few millimeters, known as Bragg’s peak [1, 2]. It is possible to deliver the dose to a tumor region with the added benefit of no exit dose [1, 2]. A neutron beam has a relatively better biological effectiveness compared to that of photon beams [3]. Recently, particle therapy techniques using boron compounds, such as the boron neutron capture therapy (BNCT) method, have been progressed to improve therapeutic effects in particle radiation therapy [4–6]. Moreover, a prompt gamma ray induced by a reaction between the particle and boron can provide prompt gamma ray image during treatment [7, 8]. In particular, proton boron fusion therapy (PBFT) has been suggested as a novel radiation therapy technique and tumor monitoring technique for use during treatment. The PBFT method is a treatment technique based on the proton-boron fusion reaction [6, 8]. The proton captures 11-boron (^11^B), resulting in three alpha particles (one 3.76 MeV, two 2.46 MeV) and a 719 keV prompt gamma ray [6, 8]. Theoretically, as BNCT generates one alpha particle after the final reaction, the therapeutic effect of PBFT per incident particle is three times greater than that of BNCT [9]. In addition, based on data from our previous studies, we confirmed that the therapeutic effect of PBFT can be improved by as much as 1.5 times compared to that of a proton beam without a boron compound [6, 8]. We also confirmed the generation of a 719 keV prompt gamma ray after the proton-boron reaction. Thus, it was possible to develop a tumor-monitoring technique using a prompt gamma ray during PBFT. The main benefit of this prompt gamma ray imaging technique is a tumor monitoring method that does not require an extra dose during treatment, unlike computed tomography (CT) and X-ray. We can verify the boron distribution in the patient’s tumor region during proton therapy using a nuclear medicine imaging device. Because the imaging method is based on gamma ray detection, it is not a mean the real-time imaging technique. However, we can sparsely obtain an idea of the transition of the tumor and boron distribution during particle beam treatment through the method of the nuclear medicine imaging technique.

In our previous studies, a prompt gamma ray image was acquired with a specific boron concentration that was much higher than the clinically appropriate boron concentration range, so those studies were more conceptual in nature [6, 8–10]. In this practical study, a boron concentration range allowable in clinical applications was used for prompt gamma ray imaging during PBFT. The purpose of this study is to evaluate prompt gamma ray images during PBFT with Monte Carlo simulations using the boron concentrations allowable in clinical application. This study shows the effectiveness of the prompt gamma ray imaging technique during PBFT. It can be reflected to actual imaging techniques using boron compounds in clinical applications.

## RESULTS AND DISCUSSION

**Figure 1:**
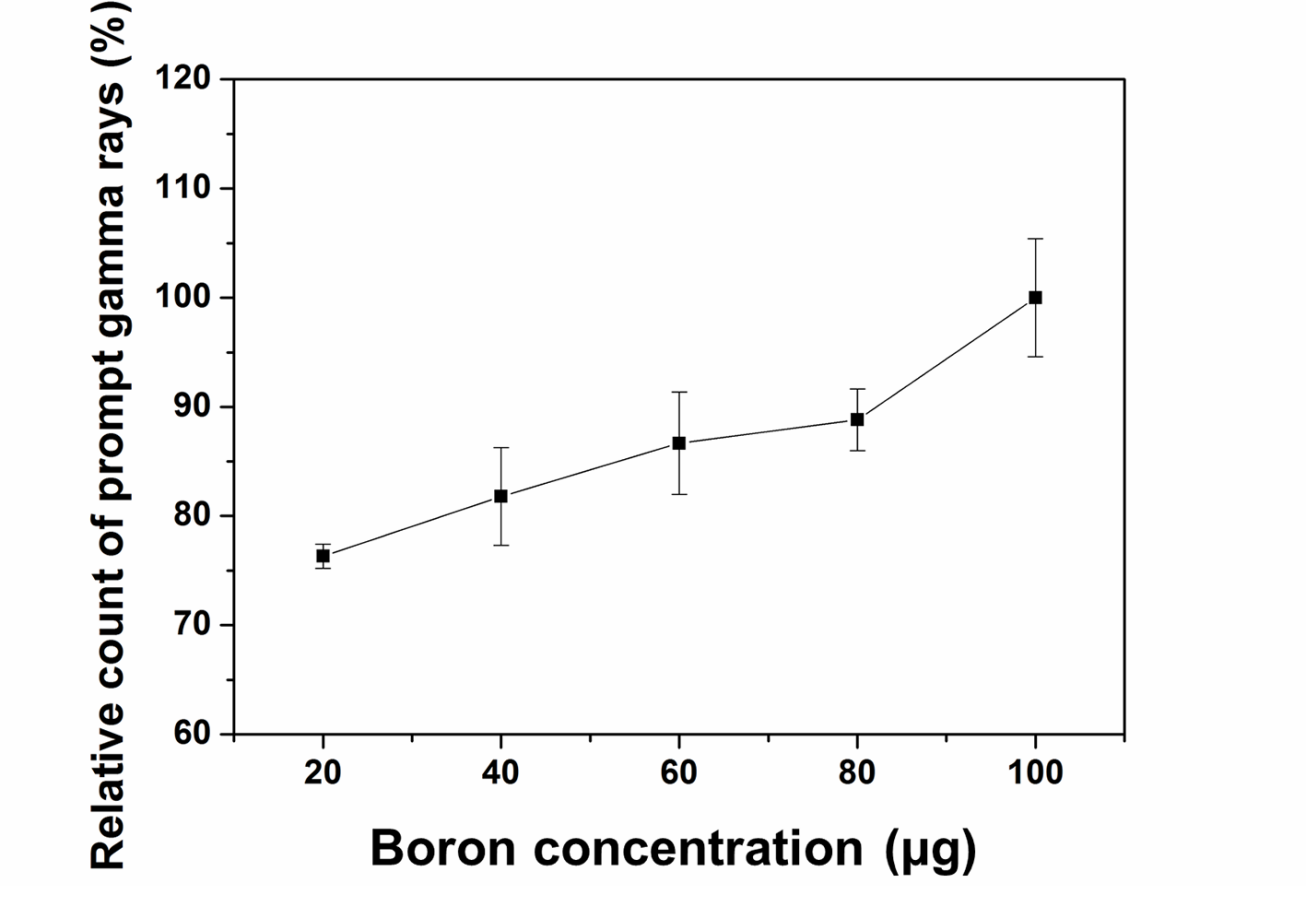
Normalized count of prompt gamma rays as a function of boron concentration The average prompt gamma ray counts are proportional to the boron concentration.

Figure 1 shows the relative prompt gamma ray counts based on the MCNPX simulation. This figure was normalized with a mean value of 100 μg at the maximum value. The number of counted prompt gamma ray events increased with increasing boron concentration. This means that a higher boron concentration causes a higher number of 719 keV prompt gamma rays to be generated by the proton boron fusion reaction.

**Figure 2:**
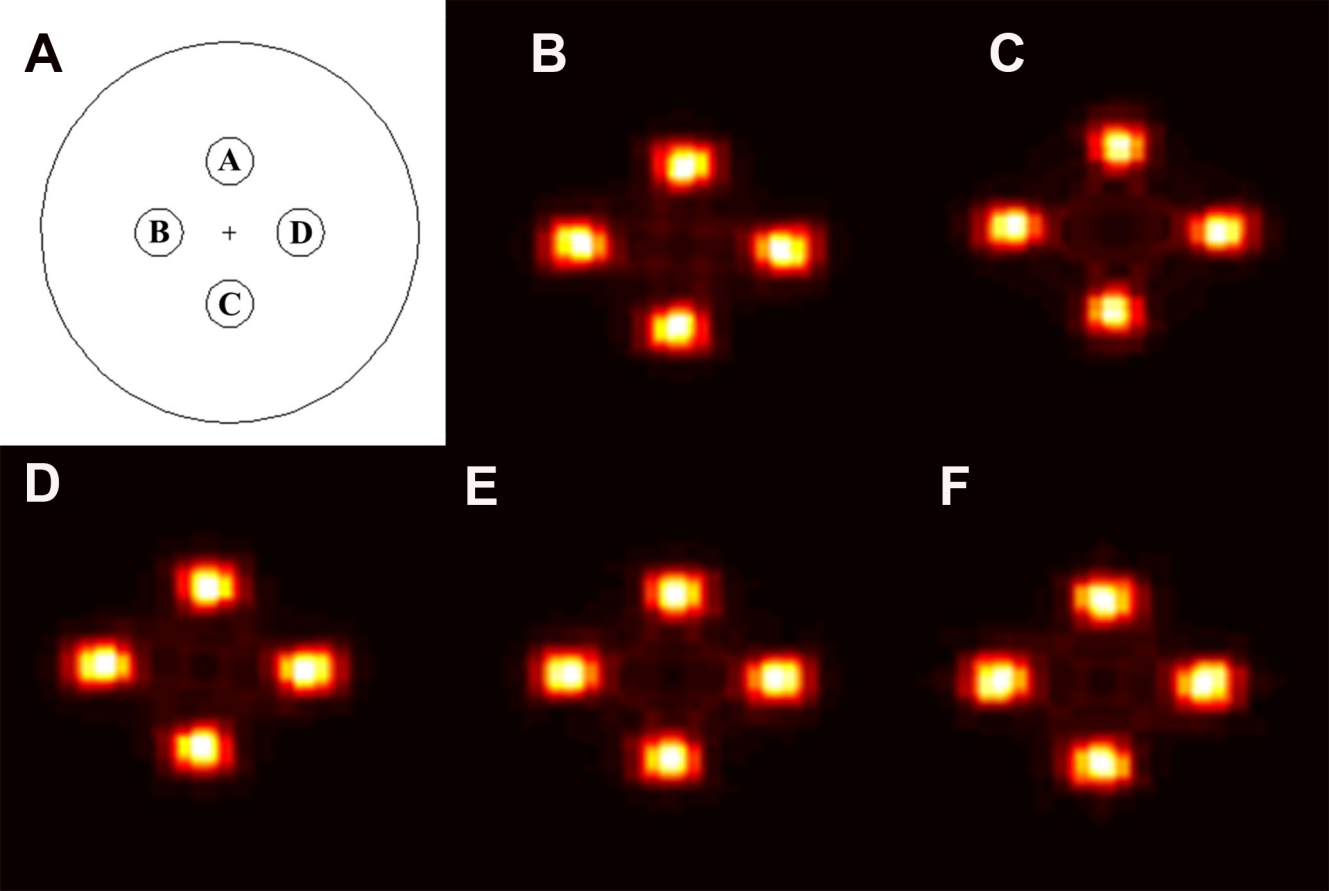
(**A**) Original pattern (axial view) of the virtual water phantom including the four boron uptake regions (BURs). This diagram indicates the location and geometric specifications of each BUR with the same diameter in the virtual water phantom. Prompt gamma ray images are shown in (**B–F**) at boron concentrations of (B) 20 μg, (C) 40 μg, (D) 60 μg, (E) 80 μg, and (F) 100 μg.

Figure 2 shows the original pattern (axial view) of the virtual water phantom and the reconstructed images using prompt gamma ray events with different boron concentration. The prompt gamma ray events were sorted using particle tracking data, which details every event such as collisions and scattering in the MCNPX. Although the number of counted prompt gamma ray events at 719 keV varied with the boron concentration as shown in Figure 1, there was no significant difference between each reconstructed image as shown in Figure 2B–2F. Although the concentration of 80 µg/g is twice of 40 µg/g, the difference in the average relative count in only 5%. Moreover, for some cases, a higher count was achieved for the 40 ug/g case than 80 µg/g. The reconstruction time for (b), (c), (d), (e), and (f) was 4.27, 4.43, 4.60, 4.63, 4.92, respectively. For these reasons, a quantitative analysis of the prompt gamma ray images for each boron concentration was required. We focused on analysis of the image profile, the SNR, the contrast, and volume difference between the physical target volume and prompt gamma ray image volume in the BURs using prompt gamma ray images for each boron concentration.

**Figure 3:**
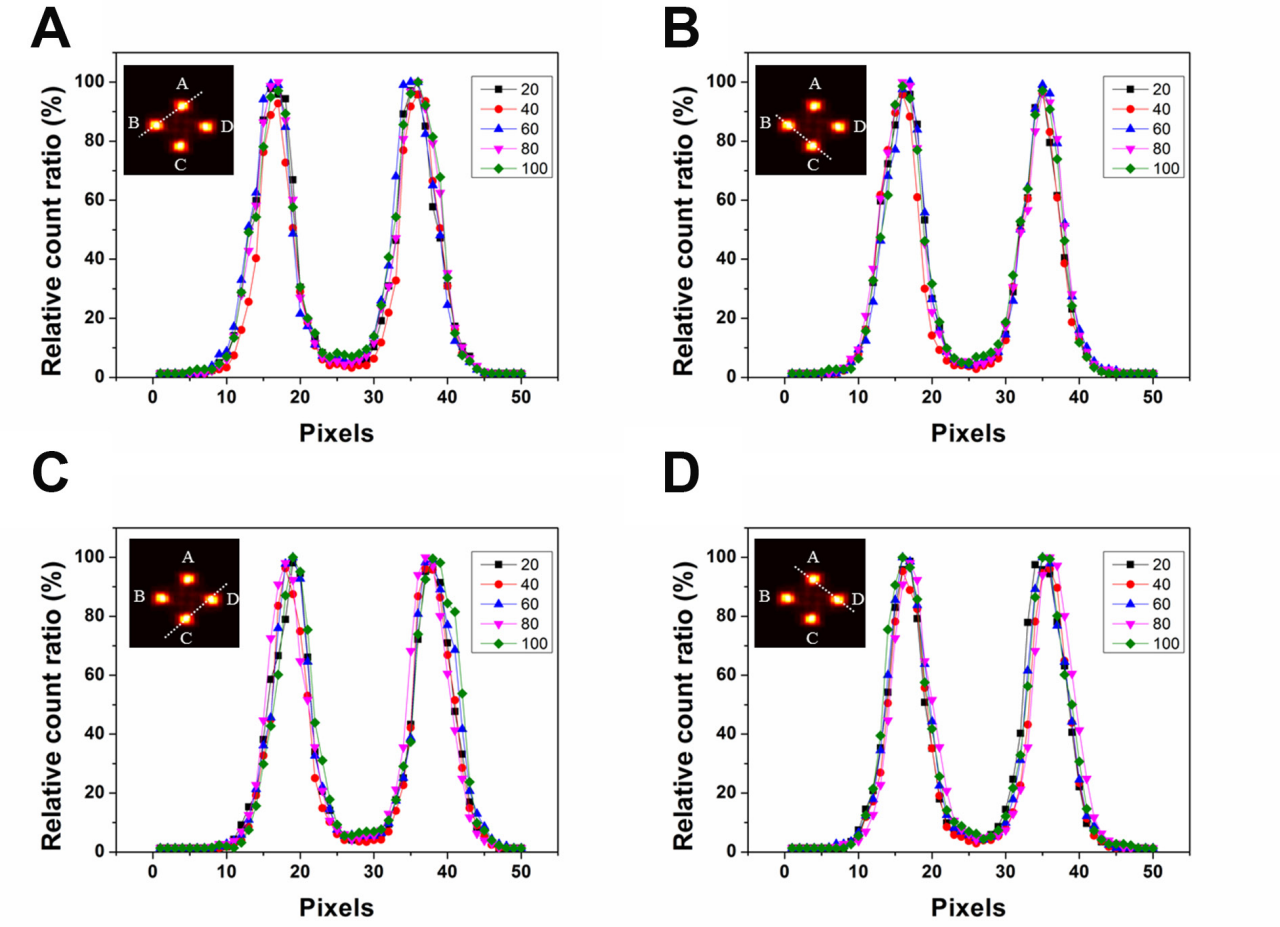
Image profiles over two boron uptake regions (BURs) for various boron concentrations The y-axis shows the relative count ratio of the profile of the prompt gamma ray image.

As shown in Figure 3, each BUR was clearly distinguished from the background region in each prompt gamma ray image. After we acquired the image profiles across the white dot line as shown in Figure 3, we performed the normalization of the image profiles using the highest peak value for each case. Although the standard of normalization for cases (a), (c), and (d) was 100 µg/g, for case (b), a 60 µg/g peak value was used as the standard of normalization.

Because we used the same geometry for the four BURs to simulate a phantom, they each showed a similar signal intensity and pattern on the image profile. Moreover, when we observed the image and profiles with naked eyes, the difference was difficult to identify. For this reason, we analyzed both the SNR and contrast value rather than full width at half maximum (FWHM) value for each image [11].

To compare prompt gamma ray imaging performance across boron concentrations, the SNR and contrast were calculated from each image, as shown in Tables 1 and 2. To calculate the SNR and contrast, the size of the ROI was adjusted to the value of BUR’s physical diameter, which is extracted from MCNPX code [5]. As this phantom has four regions, we set four ROIs on the four circular regions and one ROI on the background region [8]. The four ROIs were arranged to coincide with the four BURs. To evaluate the noise effect, the ROI for the background was set to the diameter of the BUR and was located at the center of the water phantom, which did not include any BURs. As shown in Figure 3, the intensities of the image profiles showed a similar tendency as a function of boron concentration. However, the SNR improved slightly with increasing boron concentration, as shown in Table 1. The contrast value also slightly improved as a function of the boron concentration. In addition, we confirmed that if the boron concentration is increased, the difference between the target volume and the volume of the prompt gamma ray image at the BUR decreased as by the following amounts: 20 μg: 30%, 40–80 μg: 14%, 100 μg: 11%. As a result, higher boron concentration resulted in higher prompt gamma ray image reproducibility. Because a new boronate compound is being developed to increase the boron uptake ratio in the target volume, the reliability and usefulness of prompt gamma ray images can be gradually increased [12, 13]. Thus, the clinically applicable boron concentration range can also be increased. A higher boron concentration can induce a smaller irradiation time. Because there are more boron particles in higher boron concentration, more alpha particles are naturally generated. The damage level to the tumor cell will increase according to the number of the alpha particles. Therefore, because a large dose equivalent can be satisfied by using small irradiation, the treatment time can be reduced. Moreover, there were no significant differences among the prompt gamma ray images as a function of boron concentration as viewed with the naked eye, as shown in Figure 3. These results suggest that tumor monitoring via the prompt gamma ray detection can be clinically applicable even if the boron concentration is relatively low.

**Table 1:** Signal to noise ratio (SNR) values for each boron uptake region (BUR) at various boron concentrations

Boron concentration (μg)	A	B	C	D
20	27.31	28.72	27.57	28.81
40	30.84	31.92	30.84	31.65
60	32.24	33.25	31.84	33.25
80	32.26	33.62	32.26	33.62
100	33.70	35.18	33.71	35.18

**Table 2:** Contrast values for each boron uptake region (BUR) for various boron concentrations

Boron concentration (μg)	A	B	C	D
20	0.68	0.69	0.68	0.68
40	0.71	0.73	0.72	0.73
60	0.74	0.75	0.74	0.75
80	0.75	0.76	0.75	0.76
100	0.76	0.77	0.76	0.77

The proposed prompt gamma ray imaging technique in clinical fields is useful to observe the tumor status under treatment during proton boron fusion therapy. Because the generation point of prompt gamma ray induced by the proton boron fusion reaction can be demonstrated in the image as a hot spot, we can recognize the treatment status of the tumor. If the hot spot is identified at the incorrect point in the image, it is possible to can stop the treatment immediately. Further, if an over-dose or under-dose scenario is identified from the image, we can correct the treatment plan, patient positioning, boron concentration, etc., to improve the quality of therapy.

## MATERIALS AND METHODS

**Figure 4:**
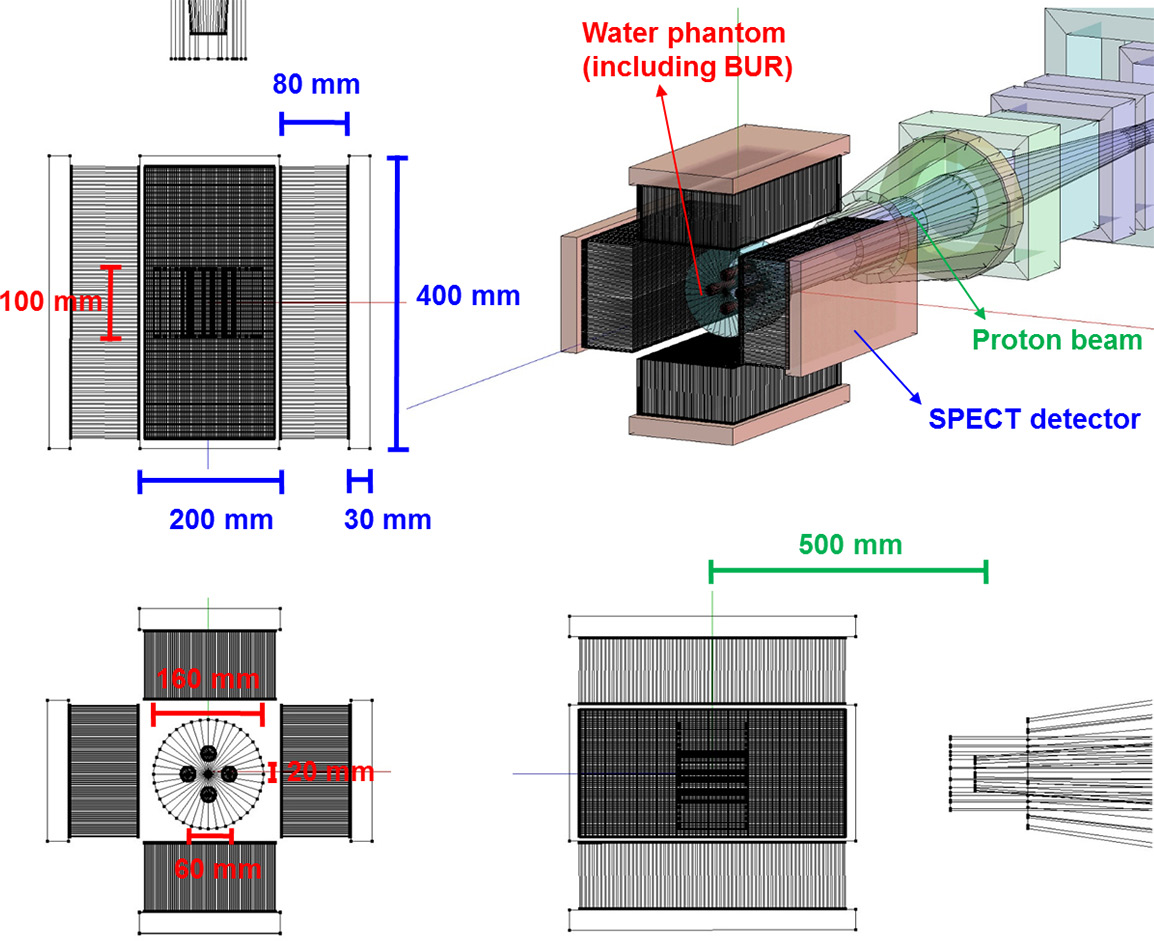
Schematic diagram of simulation for prompt gamma ray imaging using boron during proton irradiation There are cross-sectional, ground, elevation diagram and three dimensional pictures of simulation geometry. The water phantom has a height of 100 mm and diameter of 160 mm with four boron uptake regions (BURs; diameter = 20 mm, height = 100 mm). Size of SPECT detector was 400 × 200 × 30 mm^3^. The distance between the center of phantom and the source was 500 mm.

To assess prompt gamma ray imaging as a function of boron concentration for PBFT, the therapeutic conditions of PBFT were set using Monte Carlo n-particle extended simulation code (MCNPX, Ver. 2.6.0, National Laboratory, Los Alamos NM, USA), as illustrated in Figure 4 [14]. For the simulation, a cylindrical virtual water phantom including boron uptake regions (BURs) was simulated. Although we simulated the phantom focused on the brain case, our simulated phantom is too simple to show the function of the real brain phantom. Originally, because we constructed the phantom for easy comparison of only imaging, more detailed construction is needed for the phantom. This cylindrical virtual phantom (material = water, diameter = 16 cm, height = 10 cm, density = 1 g/cm^3^) included four BURs with a cylindrical pattern (diameter = 2 cm, height = 10 cm), and the boron concentration was 20 to 100 μg at intervals of 20 μg (20, 40, 60, 80, and 100 μg) because this boron concentration range has been widely used for BNCT in actual clinical applications [4, 5, 15, 16]. Actual boron uptake to tumor induces the background of the small amount at the around the tumor. The uptake ratio between the tumor and around normal tissue (nearby blood vessel) was 1:4–5. It can be changed according to the kind of the boronate compound, physiological conditions, etc. However, because the detection efficiency is very low for prompt gamma ray detection, we expect that the effect of the background to be negligible. Therefore, in this study, we did not set the background of boron around the target regions. The center locations of the four BURs were A: (0 cm, 0 cm, 3 cm), B: (−3 cm, 0 cm, 0 cm), C: (0 cm, 0 cm, −3 cm), and D: (3 cm, 0 cm, 0 cm), as shown in Figure 2A. The physics model in MCNPX can be applied by using the “physics function” in the code. In the physics function, we can set the eight factors as follows: particle type, upper energy max, analog energy limit, table-based physics cutoff, unused placeholder 1, controls charged-particle straggling, unused placeholder 2, light-ion recoil control. Because the proton energy was set to 80 MeV, the upper energy limit was set at 100 MeV in order to prevent the unexpected energy variation. The remaining seven factors are set at default settings. The number of proton particle in one projection was 600 million. The total number of proton particles was 38.4 billion. The statistical uncertainty per one reaction was demonstrated using the P-trac data, which is one of the results of the MCNPX simulation. The average statistical uncertainty was below 0.01%. We used an 80 MeV proton beam, and the distance between the source and the center of the phantom was 50 cm [6]. This beam conditions were based on the simulation conditions from previous study [6]. We focused on variation of the image as a function of the boron concentration. In order to confirm the variation in the image, we should change at least one condition (reaction-cross section, detector material, reconstruction algorithm, etc.). In this study, we changed the boron concentration. Although we can confirm the variation in the number of effective events from the projection, it is difficult to determine a visible difference in the prompt gamma ray image (nuclear medicine image). Originally, the nuclear medicine image can be reconstructed by the difference between the signal’s level and noise’s level. If the tendency of the relative count of prompt gamma rays is almost the same with the other energies, the variation trend of image is almost the same with the other energies. Thus, we chose a representative beam case that could show the difference as a function of the boron concentration.

Our previous study investigated imaging using a 719 keV prompt gamma ray during PBFT via simulation [8]. In the previous study, a four-head single photon emission computed tomography (SPECT) scanner was simulated for imaging. To investigate only the image variations, all simulation conditions were referred such as SPECT geometry, detection conditions, beam setting, and imaging conditions [6, 8]. For the four-head SPECT scanner, Lutetium-yttrium oxyorthosilicate (LYSO, density: 7.2 g/cm^3^) was used as the scintillator material, and a 400 × 200 × 30 mm^3^ module was designed to be attached to the parallel collimator [17]. The high energy parallel collimator material was tungsten (density = 17.3 g/cm^3^, thickness = 0.25 cm, the number of holes = 67, hole size = 0.35 cm, and height = 8 cm). Because the distance between the collimator and the phantom was 2 cm, this detection structure was configured to have a high detection efficiency. For a tumor monitoring device, a high detection efficiency prompt gamma ray and fast image reconstruction algorithm are essential for clinical applications. Therefore, we used the ordered subset expectation maximization (OSEM) using a graphics processing unit (GPU) for fast image reconstruction. This algorithm which computed unified device architecture (CUDA) has been used several times in previous studies [18].

In order to acquire the result in Figure 1, we simulated each boron concentration case 10 times. When we make the simulation code, the random function “dbcn” in MCNPX was used. Because this function can generate independent random variables for the simulation, even when the same code is simulated, slightly different results were obtained with below 0.01% error. For each boron concentration case, we repeated the simulation. We obtained an average value from 10 results. And we added the standard deviation value at each result as the bar pattern. Prompt gamma ray images were reconstructed using a 285 × 285 domain matrix with a pixel size of 0.3 mm. The modified OSEM reconstruction method was performed using eight subsets and five iterations. To reduce the computation time for image reconstruction, parts of the domain were assigned to each subset calculation without overlap [18]. In our reconstruction process, there is no pre-process that includes filtering. Because the EM algorithm can be operated according to the maximum likelihood, the probability variables are applied to the image. In order to increase the image reconstruction speed using the EM algorithm, we have to divide the subset for the maximum likelihood or accelerate the computation power. Although the conventional OSEM has several subsets for the maximum likelihood, the results of the reconstruction are almost same with those of MLEM. In our previous study, to reduce the image reconstruction time, we used the GPU-based computation method [18]. For the GPU system, there are several threads that can perform computations individually. For heavy calculations, if each thread can perform individual calculations simultaneously, the heavy calculation can be finished within a short time. Thus, we divided the computation according to the number of threads in the GPU system for reconstructing the image. In order to divide and assign the calculation, the OSEM algorithm equation should be changed. First, an assignment algorithm for the thread and a partial reconstruction algorithm for the assigned thread are required. Second, the computation results collected by the algorithm for each thread should be applied to the equation. In this manner, we can achieve the final equation of the modified OSEM for the application of the GPU computation.

With this modified reconstruction algorithm that uses a GPU, we can acquire the prompt gamma ray at very fast speeds [18]. However, the time for image acquisition is limited to the treatment time. To acquire at least two images during a treatment session, we must find an image acquisition method that uses a low projection number and has a short computation time. In this study, we used 32 projections having 11.25°. Because the SPECT consisted of four modules, only eight projections per head module were performed. To acquire effective prompt gamma ray events for imaging, the energy window was set to 20 % of the 719 keV peaks. This value is a general setting when a gamma ray is detected with the gamma camera [19, 20].

For quantitative analysis of prompt gamma ray imaging as a function of the boron concentration, we sorted the events the prompt gamma ray events into energy windows at 719 keV from the particle tracking data in MCNPX. The number of prompt gamma rays at 719 keV is related to the efficiency of the reaction between the proton and boron as a function of the boron concentration. In principle, although a higher number of events provides a clearer prompt gamma ray image, an insufficient number results in a poor image. Moreover, the number of events is limited by the treatment time. Thus, we attempted to overcome this problem using the modified OSEM reconstruction algorithm.

After acquisition of a prompt gamma ray image, the image profile was measured drawn through two BURs as a function of boron concentration. To compare the level of signal from the BUR and background noise, the contrast and signal-to-noise ratio (SNR) were acquired for the region of interest (ROI) for each BURs and for the background. The contrast and SNR can be calculated using the following equations: [21, 22]

$$\mathrm{Contrast}=\frac{\left|S-B\right|}{S+B}$$

$$\mathrm{SNR}=\frac{S}{B_{\mathrm{SD}}}$$

where *S* is the average signal in the BUR, *B* is the average background signal, and *B*_*SD*_ is the standard deviation of the background. The size of the ROI was the original physical diameter of the center of the BURs based on the MCNPX input source code. As this phantom has four regions, we set four regions of interest (ROI) on the four circular regions and one ROI on the background region. The size of the ROI was set at 100% of the physical inner diameter.

To assess the correlation between the physical phantom used in the simulation and the prompt gamma ray image, an analysis of the geometric accuracy was performed using the percentage difference of the volumes, which is a ratio between the volume of the prompt gamma ray image and the target volume of the physical phantom. The target volume of physical phantom was extracted from the MCNPX input source code, and the volume of the prompt gamma ray image was superimposed on the original pattern of the virtual physical phantom using the pixel matching method with a threshold. The threshold value was set to 10% of the maximum signal intensity because the background signal intensity was less than 10% of the maximum signal in the BURs.

$$\mathrm{Percentage}\;\mathrm{difference}\;\mathrm{of}\;\mathrm{volumes}=\frac{V_{\mathrm{PT}}-\mathrm{TV}}{\mathrm{TV}}\times 100(\%)$$

where *V*_*PT*_ is the volume of the prompt gamma ray image and *TV* is the target volume of physical phantom used in the simulation.

## CONCLUSIONS

In this study, we analyzed the prompt gamma ray images according to the boron concentration during the proton boron fusion therapy, using the Monte Carlo simulation. However, because this study was conducted using only Monte Carlo simulation, this methodology could not directly prove the actual clinical effectiveness. However, in this study, we considered the actual clinical use of boron concentration for acquiring the prompt gamma ray image. In addition, this study focused on the physical evaluation of the acquired image according to the boron concentration. The results of this study show that tumor monitoring during treatment is possible using prompt gamma rays at 719 keV, even if the boron concentration is not high. In future studies, we will perform a phantom study and pre-clinical study using a gamma camera with a high spatial resolution, which can distinguish scatter radiation even under 1 MeV during PBFT.
